# Coloniality, Elite Networks and Intersectionality: Key Concepts in Understanding Biomedical Power and Equity in Health Policy Processes: Comment on "Power Dynamics Among Health Professionals in Nigeria: A Case Study of the Global Fund Policy Process"

**DOI:** 10.34172/ijhpm.2023.7916

**Published:** 2023-04-15

**Authors:** Rakesh Parashar, Veena Sriram, Sharmishtha Nanda, Frayashti Shekhawat

**Affiliations:** ^1^Health Policy and Systems, Sambodhi Research and communications, Noida, India; ^2^School of Population and Public Health, University of British Columbia, Vancouver, BC, Canada; ^3^School of Public Policy and Global Affairs, University of British Columbia, Vancouver, BC, Canada; ^4^Independent Researcher, New Delhi, India; ^5^Global Studies Ambedkar University, Delhi, India

**Keywords:** Power Analysis, Power in Health Policy, Colonialism, Intersectionality, Health Policy Analysis, Biomedical Hegemony

## Abstract

To understand the role of power in health policy processes in low- and middle-income country (LMIC) contexts, it is necessary to engage with global and local power structures and their historical contexts. In this commentary, we outline three dimensions that shape a dominant power in health policy processes—the biomedical power. We propose that understanding the linkages between medical power and colonialism; the close connection of public health, medicine and elite networks; and the intersectionalities that shape the powers of medical professionals can offer the means to examine the biomedical hegemony in health policy processes. Additionally we suggest that a more nuanced understanding of the interaction of local powers with global funding can offer some entry points to achieving more equitable and interdisciplinary health policy processes in LMICs.

## Background

 In their case study from Nigeria, Lassa and colleagues^1^ show how the interactions of global and country-level policy processes can sustain professional monopolies in policy-making, rendering policy process reductionist and perpetuating local inequities. Examples of how existing professional hierarchies allow medical practitioners to actively dominate and ‘drive’ the policy agenda even on non-clinical matters that may require the expertise of social scientists, implementers or patients themselves, holds true in many country contexts.^2^ The study brings up a useful message that theories from the sociology of professions can help understand biomedical dominance in health policy-making. While we resonate with the authors, we also argue that addressing the issue of biomedical hegemony in global funding and local policy-making requires engaging with several other aspects. For instance, it is critical to unravel the historical patterns in global health and understand the close relationship between modern public health and clinical medicine. These relationships are also closely linked to the dominant, industry-sponsored funding which has traditionally favoured biomedical thinking in health policies, contributing to disproportionately higher say of medical professionals. Likewise, as Lassa and colleagues show that clinicians used a biomedical discourse in Nigeria to shape the Global Fund proposals, the epistemic and normative powers of medical professionals in society and the organisation of health services make it further challenging to deploy interdisciplinary approaches in health policy-making.^3^

 Building on the central issue in the study by Lassa and colleagues — the power of medical professionals in the health policy process — we outline three dimensions that are useful to engage with the issue further. First, we touch upon the role of colonialism in bolstering biomedical hegemony; Second, we outline the continued, dominating connection between medicine and public health; and in the third dimension, we bring up the point about the heterogeneity in the powers of medical professionals, shaped by several intersectionalities.

###  Medical hegemony (and dominance of biomedically driven funding) in health policy and programming has its roots in the ‘professional project’ accelerated by colonialism

 Lassa et al observe that “occupational hierarchy places medical professionals as the head of health units in the public sector, which other health occupations appear to have internalised as the norm,” an insight that rings true in South Asian contexts. The dominance of medical professionals in healthcare is global, but it takes a particular shape in many low- and middle-income countries (LMICs) due to the imprint of colonialism, as noted within the commentary. The shared history of colonialism across many countries is indeed one factor in explaining why medical professionals hold such strong institutional power — from high-level policy-making to facility management — and also provides insight into the complexities of addressing such power imbalances. Scholars have presented a rich picture of the ways in which colonial powers developed “formal” health systems around biomedicine side-lining traditional systems of medicine, particularly from policy-making, despite the vast utilisation of traditional medicine by the public (a trend which continues to the present day). In combination with an approach to biomedicine that prioritised medical services catering to urban elites and “disease control” (epidemic management) for rural communities, this framework resulted in biomedical paradigms dominating health decision-making, rather than more holistic approaches to health that integrate primary care, community-based approaches and broader social determinants of health (a trend noted by Lassa and colleagues). One such example is the history of malaria control in the African region. European colonisers had initiated malaria control efforts in several African countries, primarily to protect their own troops and to maintain their economic interests.^4^ As a result, biomedical interventions, such as malaria treatments and insecticide sprays, were prioritized for many decades, with limited consideration of social determinants, as well as the harmful impact of insecticides on the environment.

 Post-colonial governments’ goals around modernity and scientific pursuits further emphasised the role of biomedicine in newly independent nations. Within this biomedical framework, the role of doctors was pronounced.^5,6^ In India, a clear example of this is evidenced by efforts of physicians to dismantle cadres of non-physicians clinicians, known as Licentiate Medical Practitioners, and by the continued challenges in expanding the scope of medical practice to other cadres, such as nurses, traditional practitioners and other practitioners.^7^ The power of medical professionals is visible across many contemporary debates in health policy and systems, including challenges pertaining to the scope of practice and the regulation of provider behaviour.^8^

 The trends outlined by Lassa et al are therefore a continuation of longstanding historical patterns that situate doctors at the “top” of a biomedical hierarchy and that have traditionally situated biomedicine above public health, traditional systems of medicine and other approaches to health. That said, there is evidence that these old patterns are evolving into something more complex, with the growing politicisation of debates between biomedicine and traditional forms of medicine and an unsettling of medical power during the COVID-19 pandemic.^9^

###  The close connection of public health with medicine is sustained by elite networks which also mediate the translation of global funding to local programs

 Lassa and colleagues interestingly raise the term ‘public health doctors,’ referring to medical doctors who have received public health training. This term addresses the linkages between medicine and public health and complicates the narrative of medicine and public health as distinct siloes. In fact, public health training is a pathway sought by many doctors seeking to broaden their impact on public health and health services. The ways in which such training is diverse, with individuals utilising their public health training in a variety of avenues in research, practice and teaching, and across multiple sectors (private for-profit, private not-for-profit, and the public sector).

 There is one aspect of the nexus between medicine and public health that is particularly salient to the issue of medical dominance; public health training might serve to legitimise actions taken by doctors that prioritise biomedical interventions over broader public health actions. Specifically, the insights raised by Lassa et al on the use of biomedical language also suggest that the biomedical discourse also included epidemiology (the use of the term “Epi-analysis”), indicating that medical experts with public health training have a particular advantage in drawing upon multiple domains of knowledge. As noted earlier, doctors with public health training might use public health training in a variety of ways, but one avenue might be the direction taken by the medical doctors observed in global health initiatives at the national level, such as the processes of the Global Fund observed in Nigeria by Lassa and colleagues.

 The role of elite schools of public health in bolstering the linkages between medicine and public health, including in the context of global health initiatives, warrants further attention. Networks are well recognized as important mechanisms of idea diffusion in global health,^10^ and the networks forged by schools of public health internationally have historically been important conduits for global health policy. However, schools of public health might also counteract the discussed power imbalances by several means, such as fostering diversity and inclusion, encouraging critical thinking, and engaging with dominant narratives in global health, and engaging with communities and social-political determinants of health.

###  The powerful influence of medical professionals on health policies is not homogenous, and several intersectionalities shape their powers

 Lassa et al aptly argue that the relative monopoly of medical professionals in the policy process creates obstacles to multidisciplinary policy-making. In this context, it is critical that these processes have adequate representation of social, and political scientists, implementers, and diverse forms of health workers- especially the ones at the front lines. However, the issue of representation can be better understood by the intersectionalities that shape the powers and representation of medical professionals in health policy-making.^3^ Medical professionals belong to several social layers in different country contexts. These layers could be related to hierarchies such as their ranks and experience in the health systems, place of work etc and to social structures such as caste, class, gender, religion etc.^11^ For instance, normative gendered power relations are likely to shape the representation of female professionals in clinical and policy-making settings. Existing research points to the low numbers of women in medical academic/administrative positions, as well as in decision-making roles.^12^ Moreover, in contexts such as India, caste-based professional and economic kinship networks have accorded privileges to certain groups over others, and the colonial introduction of modern medical training in India did not disrupt the pattern. High-caste men and (some) women, even within the medical profession, hence, still retain the position of power elites owing to generational access to English education and technical education.^6,13^ Some of these intersectionalities were evident during the COVID-19 response in India and several other countries, where the medical professionals belonging to clinical specialities, industry and professional associations had higher influence in government-run task forces compared to public health specialists from other backgrounds.

 With several intersectionalities underpinning the powers of medical professionals, and their linkages with globally funded policies and programs, we propose that studying the differential powers of medical professionals can provide a potential route to examine the pathways for biomedical dominance and can help in building interdisciplinary approaches in health policy-making.

## Conclusion

 The above dimensions could aid in advancing the knowledge about the role of biomedical powers in national and subnational health policy-making processes. At the same time, a more nuanced understanding of the interaction of local powers with global funding can offer some entry points to achieve the goal of the equitable and interdisciplinary health policy process in LMICs. Keeping this goal in mind, we propose some additional suggestions below.

 As a majority of global health funding and agendas are accessed in the LMICs by the local elites – also termed as ‘elite capture,’^14^ it can sustain or aggravate the power imbalances.^.^ One such example is the PEPFAR (President’s Emergency Plan for AIDS Relief) program in Uganda. PEPFAR is a large-scale, multi-country HIV/AIDS treatment and prevention program funded by the US government and implemented in Uganda between 2004 and 2013. Critics of the program argued that decision-making was heavily dominated by international actors and local elites who had close ties to the Ugandan government, with limited involvement of community-based organizations and grassroots groups.

 Hence, it is important to examine all funding from a lens of whether it has the potential to reinforce the existing inequities, at national and subnational levels. One of the approaches to ensure this could be to pass the funding through equity frameworks applicable to various national and subnational contexts and the concerned policies. Scholars have called to use ethical frameworks and principles such as equity, equality, diversity and inclusion in global health funding, and related implementation and research projects earlier.^15^ For example, some recent funding approaches have mandatorily included a gender perspective in proposals, seeking to promote gender equity in health programming, however, it is unknown whether and to what extent such checklists translate to gender-equitable programming and research. Nonetheless, we suggest that global funding and related local policy processes, identify and address the potential drivers of inequity by using contextually adopted equity frameworks. Since some of these frameworks are available and applied already, it is critical to articulate the relevant equity issues in activities done under each funding cycle and go beyond simplistic considerations of equity. For example, while addressing low coverage of certain medical technologies or pharmaceuticals in specific geographies, it is crucial to consider and address the social, economic and political realities of marginalized populations that exacerbate these outcomes.

 Likewise, there is a need for sustained, meaningful engagements with global funders, national and subnational policy-makers and implementers in order to promote interdisciplinary and equitable policy thinking. It is important to build more contextual evidence, as Lassa and colleagues do, and to offer clear examples of how global health funding-supported policies sustain or aggravate existing inequities in local contexts. Demonstrating such evidence can be partly achieved by more locally led research focused on the policy-making stage, including examining the dominant actors and their powers.

 At the same time, it is also important to build dialogue across stakeholders such as funding bodies, policy-makers, researchers, implementers and the public to reflect on the potential of contextual injustice the globally funded programs can inflict, and how this can be addressed.^14^ Effective advocacy in this direction could also be done by presenting evidence (such as the study from Lassa and colleagues) on how engaging neglected actors, such as frontline and community voices, can enhance interdisciplinary and equitable engagement with policy.

## Acknowledgements

 We thank the *International Journal of Health Policy and Management* editors to invite us for this commentary.

## Ethical issues

 Not applicable.

## Competing interests

 Authors declare that they have no competing interests.
